# Abdominal Wall Endometriosis in Appendectomy Scar 42 Years After Initial Surgical Procedure—Case Report and Literature Review

**DOI:** 10.3390/reports9030216

**Published:** 2026-07-09

**Authors:** Thomas Ferenc, Darko Blašković, Karolina Krstanac, Mislav Rakić, Mateja Vujica Ferenc, Vinko Vidjak

**Affiliations:** 1 Department of Diagnostic and Interventional Radiology, Merkur University Hospital, 10000 Zagreb, Croatia; darko.blaskovic@gmail.com (D.B.); vinko.vidjak@gmail.com (V.V.); 2 Department of Pathology, Dubrava University Hospital, 10000 Zagreb, Croatia; kkrstanac@kbd.hr; 3 Department of Abdominal Surgery, Dubrava University Hospital, 10000 Zagreb, Croatia; mislav78@gmail.com; 4 School of Medicine, University of Zagreb, 10000 Zagreb, Croatia; 5 Department of Obstetrics and Gynecology, University Hospital Centre Zagreb, 10000 Zagreb, Croatia; matejavujica1@gmail.com

**Keywords:** abdominal wall endometriosis, suture granuloma, Schloffer tumor, ultrasound, Doppler ultrasound, elastography, contrast-enhanced ultrasound, contrast-enhanced computed tomography

## Abstract

**Background and Clinical Significance**: Abdominal wall endometriosis (AWE) is an ectopic endometrial tissue embedded into the anterior abdominal wall, mainly infiltrating the rectus abdominis or oblique muscles and subcutaneous tissue. In most cases, AWE is associated with surgical scars after obstetrical and gynecological, as well as non-gynecological surgeries. **Case Presentation**: A 51-year-old female patient presented to the ultrasound outpatient clinic with a non-cyclic painful, palpable nodular mass located in the postoperative scar in the right lower abdominal quadrant. She underwent an appendectomy at the age of 9 (premenarchal period). The patient had regular menstrual cycles, one cesarean section and two vaginal deliveries, denied any trauma to that abdominal region, and had no history of pelvic endometriosis. Her past medical history was also remarkable for left-sided breast cancer, and she was worried it could be metastasis. Following imaging evaluation, a preliminary diagnosis was a benign lesion in the post-appendectomy scar, most likely a suture granuloma, also known as Schloffer’s tumor. Fine-needle aspiration was performed, and findings were primarily suspicious for AWE. The patient was then referred to an abdominal surgeon for excision of the affected area, and subsequent histopathological analysis confirmed that the mass was AWE. **Conclusions**: Imaging findings of a mass in the abdominal wall are not pathognomonic for AWE; only histopathological examination can confirm the diagnosis. If a painful nodular mass is located adjacent to a surgical scar in a female patient, AWE should be a leading consideration in the differential diagnosis, along with suture granuloma in cases of old surgical scars.

## 1. Introduction and Clinical Significance

Abdominal wall endometriosis (AWE) is defined as ectopic endometrial tissue embedded into the anterior abdominal wall, mainly infiltrating the rectus abdominis or oblique muscles and subcutaneous tissue [1,2]. It typically manifests as a solid nodular mass, occasionally containing cystic zones due to intralesional hemorrhage [2]. Spontaneous AWE occurs in the scarless abdomen and accounts for approximately 20% of all cases [1,3,4]. However, in most cases, AWE is associated with surgical scars following obstetric and gynecologic, as well as non-gynecologic surgeries [1,4,5,6]. It has been reported that 57–92% of AWE cases develop secondary to cesarean section [3]. AWE in an appendectomy scar is a rare finding, with only a few reports describing its association with this entity [7,8,9,10,11,12,13,14,15]. Herein, we report a case of a female patient with an exceptionally delayed AWE in an appendectomy scar, 42 years after the initial surgical procedure.

## 2. Case Presentation

A 51-year-old female patient presented to the ultrasound (US) outpatient clinic in 2024 with a palpable nodular mass located in the postoperative scar in the right lower abdominal quadrant. She underwent an appendectomy in 1982 at the age of 9 (menarche at the age of 11). The mass was palpable for more than a month with progressive discomfort, tenderness, and worsening of the pain, non-correlating with the menstrual cycle. The patient had regular menstrual cycles, one cesarean section, and two vaginal deliveries, denied any trauma to that abdominal region, and had no history of pelvic endometriosis. However, the patient was concerned it could be a metastasis. Her past medical history was remarkable for a left-sided mastectomy in 2019 and a right-sided mastectomy in 2023. Left-sided mastectomy was performed due to multiple zones of flat epithelial atypia, atypical epithelial and lobular hyperplasia, a larger zone with ductal carcinoma in situ (DCIS), and microinvasive breast cancer of luminal A subtype. Level I axillary dissection was also performed. Right-sided mastectomy was preventative on the patient’s request due to positive family history for breast and gynecological cancer (three aunts with premenopausal breast cancer and mother with premenopausal endometrial cancer). After the left-sided mastectomy, she was using tamoxifen for a short period of time. During physical examination, there was a mild erythema around the postoperative abdominal scar, and the mass was fixed and painful at palpation.

B-mode US examination demonstrated an irregularly shaped, hypoechoic mass measuring 1.7 × 1.1 cm, with lobulated, partially indistinct margins and posterior shadowing. Furthermore, a hyperechoic rim was noted surrounding the mass, with a few linear hyperechoic structures within the mass that resembled suture material (Figure 1). Color and power Doppler examinations showed mild rim-like vascularization (Figure 2A,B). On strain elastography, the mass was predominantly soft, with several areas of increased stiffness at the center and periphery. The strain ratio between the zones with increased stiffness within the mass and the surrounding tissue was 4.91 (2D shear-wave elastography was not available at the time of examination) (Figure 2C).

Additionally, a contrast-enhanced ultrasound (CEUS) examination was performed to try to differentiate the mass. A high-frequency linear probe was used with a mechanical index set at 0.07. After application of contrast media (4.8 mL of SonoVue, Bracco, followed by a 5 mL saline flush), short-interval scanning demonstrated a centrally non-enhancing mass with rapid, progressive, rim-like enhancement within the first 30 s of evaluation (first microbubbles appearing at 18 s). The total scan time was 01:17 min (Figure 3). A preliminary diagnosis was a benign lesion in the post-appendectomy scar, most likely a suture granuloma, also known as Schloffer’s tumor.

Due to the patient’s history, contrast-enhanced computed tomography (CT) of the abdomen and pelvis was also performed. Within the post-appendectomy scar, the previously mentioned mass was noted, measuring 2.0 × 1.9 cm and demonstrating a similar enhancement pattern. It showed signs of infiltration of the subcutaneous tissue and possibly the rectus abdominis muscle, with mild inflammation of the surrounding fat (Figure 4). No further extension of the mass or potentially malignant focal lesions in the abdomen and pelvis was detected.

Following imaging evaluation, a fine-needle aspiration (FNA) was performed, and cytological findings were primarily suspicious for AWE. The patient was then referred to an abdominal surgeon to perform excision of the affected area. The surgeon first removed the appendectomy scar and then, underneath the rectus abdominis fascia, removed a chocolate-colored nodular mass with suture material in the center, without signs of muscle infiltration. Complete resection of the mass was achieved. Anatomical layer-by-layer closure was performed; no tension was detected during suturing, thereby eliminating the need for mesh in abdominal wall reconstruction. Subsequent histopathological analysis confirmed that the mass was AWE (Figure 5). Resected margins were free. One specimen also showed suture material with a foreign-body-type giant cell reaction. The patient was discharged from the hospital in good general condition, with a well-healed surgical wound and no signs of recurrence at 2-year follow-up.

## 3. Discussion

The reported incidence of AWE ranges from 0.03 to 3.5%, with a mean age at diagnosis of 33.2 to 35 years [3]. According to Benedetto et al. [3], the interval between prior surgery and the diagnosis of secondary AWE ranges from 3 months to 20 years. AWE in an appendectomy scar is a rare finding. A literature review of reported cases of AWE in the appendectomy scar was performed in PubMed/MEDLINE and via an advanced Google search by two researchers independently, using the keywords Endometriosis, Abdominal wall, Appendectomy, and Scar. There were no restrictions regarding language or publishing year. A list of original articles and case reports was assembled, and discrepancies between researchers were resolved by consensus. Studies appearing to meet the inclusion criteria were reviewed in full text. Until 8 June 2026, to the best of our knowledge, only nine reports were associated with this entity (five in PubMed/MEDLINE and four in Google search) and are displayed in Table 1.

In these reports, the interval between appendectomy and the onset of AWE ranged from 8 months [11] to 35 years [8]. In our case, the interval between appendectomy and the onset of AWE was 42 years. Four cases are similar to our case regarding appendectomy being performed during the premenarchal period with subsequent development of AWE in the appendectomy scar. In a report by Haser [7], the 43-year-old patient underwent appendectomy at the age of 9 with a history of regular menstrual cycles, no deliveries, and three spontaneous abortions without curettage procedures. In a report by Hofmann et al. [8], a 41-year-old patient underwent appendectomy at the age of 6 due to perforation and had a history of two normal vaginal deliveries and one abortion. In a French case [10], the patient was 28 years old and underwent appendectomy at the age of 2 (unremarkable gynecological history). In the case of Ahmad et al. [14], the patient was 35 years old, and the appendectomy was performed at the age of 8. Her past medical history was remarkable for three cesarean sections.

There are two leading theories on the development of AWE: one suggests that multipotential mesenchymal cells may undergo metaplasia into endometriosis under suitable circumstances, and the second suggests that endometrial cells may be transported to an ectopic site, forming a focus of endometriosis [11,16]. Consequently, it can be assumed that the former theory correlates more strongly with spontaneous AWE, whereas the latter correlates more strongly with secondary AWE.

Considering these theories in the premenarchal period, it appears doubtful that endometrial cells were transferred to the appendectomy scar during surgery, since functional endometrium had not yet developed. Haser [7] suggested that the transfer may have occurred during instrumental manipulation of internal reproductive organs, whereas Lamblin et al. [10] found the right tubal fimbria adhered to the peritoneal scar of appendectomy, which may explain ectopic implantation. In Ahmad et al. [14] and our case, both patients underwent cesarean sections later in life, which may support the transfer theory. However, this hypothesis remains speculative and warrants further investigation because a causal relationship has yet to be established.

Clinical manifestations of AWE are variable. Patients typically present with cyclical pain in a mass adjacent to the surgical scar, worsening during menses and intermittently between cycles [11,14,16]. They may also present with local discomfort, swelling, or bleeding of the affected area [3]. In cases of AWE after cesarean section, pain and palpable mass, if present, could be located within a maximum distance of 5 cm from the incision site [16]. In this case, the patient presented with a palpable mass in the appendectomy scar, accompanied by progressive discomfort, tenderness, and worsening of the pain, which did not correlate with the menstrual cycle. She also had mild erythema around the scar.

In the US, AWE usually presents as an irregular, heterogeneous hypoechoic nodule or mass, sometimes containing cystic areas or scattered internal echoes, and surrounded by a hyperechoic rim [2,4,16,17]. Furthermore, AWE is typically noncalcified and demonstrates vascularization on Doppler examination [16]. In this case, AWE appeared as an irregular, hypoechoic mass with partially indistinct margins, posterior shadowing, and linear internal echoes, surrounded by a hyperechoic rim. On color and power Doppler, it showed mild rim-like vascularization.

In a study by Balaban et al. [17], AWE was associated with increased stiffness on elastography in all patients. In this case, it was predominantly soft, with several areas of increased stiffness in the center and periphery of the mass, consistent with an elastography score of 2 according to Griffith [18]. In a study by De Marchi et al. [19] from 2015, the authors proposed a classification of seven enhancement patterns on CEUS for soft-tissue tumors: P1—absence of contrast enhancement; P2—enhancement only in the periphery of the lesion; P3—thin (<2 mm) and few vessels (<5/field); P4—thinner (>2 mm) and more numerous vessels (>5/field); P5—enhancement with a reticular aspect, and both thick and thin bands inside; P6—numerous vessels and inhomogeneous enhancement with avascular areas; and P7—numerous vessels with homogeneous distribution. In a 2020 study by DeMarchi et al. [20], the authors reported that, in subcutaneous soft-tissue tumors, patterns P1–5 were considered potentially benign and patterns P6–7 potentially malignant; however, there was no statistically significant difference between the groups. In this case, AWE resembled the P2 CEUS enhancement pattern, with increased contrast media uptake in the arterial phase (<20 s). In the 2020 study, De Marchi et al. [20] also classified soft-tissue tumors by their vascularization time on CEUS (arterial phase < 20 s or venous phase > 20 s). Contrast enhancement in the arterial phase was detected in 69.4% of malignant and 66.7% of benign tumors, with no statistical difference between the groups in arterial-phase vascularization (<20 s).

On contrast-enhanced CT, AWE typically demonstrates the characteristic Gorgon sign, followed by other features such as nodule/mass irregularity with ill-defined borders, isodensity relative to adjacent muscles, and moderate enhancement [1]. In addition to the aforementioned, homogeneous density and infraumbilical location are CT features significantly associated with AWE [5]. On MRI, AWE usually displays high signal intensity on T2-weighted images and isointensity on T1-weighted images (compared to muscle), with hyperintense foci indicating hemorrhage [1]. MRI helps identify the hemorrhagic content of the mass, define the depth of infiltration, and assess the integrity of the surrounding muscle tissue [2]. In our case, MRI was not performed because the clinical presentation, US and CT findings, together with FNA cytology suspicious for AWE, were considered sufficient to proceed with surgical excision. As complete surgical excision was indicated regardless of MRI findings, an additional MRI was not expected to alter clinical management or the surgical planning.

According to Mizutani et al. [12], when an abdominal wall mass develops at a surgical scar, a suture granuloma is often one of the first considerations. Although silk is seldom used for abdominal wall sutures nowadays, in cases with old surgical scars, pathologic conditions due to retained foreign material should be considered [12]. Therefore, the differential diagnosis of AWE is broad and, in addition to suture granuloma, includes hematoma, abscess, desmoid tumor, sarcoma, hernia, or metastatic cancer [1,16,21].

Despite imaging findings suggestive of a possible suture granuloma, an FNA cytology was performed in this case to reduce the patient’s concern and exclude breast cancer metastasis. Cytological findings were primarily suspicious of AWE. According to studies by Medeiros et al. [22] and Chatterjee et al. [23], FNA cytology can be used as a rapid and accurate diagnostic option in cases of diagnostic dilemma for AWE. FNA cytology diagnosis of endometriosis is based on the presence of any two of three components: sheets of epithelial cells, stromal cells, and a variable number of hemosiderin-laden macrophages [22,23]. Definitive diagnosis was established by histopathological examination of the excised lesion.

Surgical excision is the only curative treatment option for AWE [3], and only histopathological examination of the specimen can confirm the diagnosis [10]. Surgical excision was also performed in this case without the need for mesh during abdominal wall reconstruction. Nodules greater than 5 cm in diameter that infiltrate the aponeurosis usually require abdominal wall reconstruction with a mesh [3]. Small nodules infiltrating aponeurosis, nodules infiltrating only subcutaneous fat, and nodules infiltrating below the aponeurosis (rectus abdominis muscle with or without peritoneum) usually do not require mesh [3]. Recurrence is unlikely in AWE lesions that have been completely surgically removed [2]. Recently, studies have shown that cryoablation may be a promising minimally invasive treatment option for patients with AWE [24].

## 4. Conclusions

AWE can be spontaneous or more often secondary. Patients usually present with cyclical pain in a solid, nodular mass, occasionally with cystic areas, adjacent to the surgical scar, which worsens during menses and intermittently between cycles. AWE is frequently missed because it may present years after the surgical procedure and may not always present as a palpable mass. The imaging findings of a mass in the abdominal wall are not pathognomonic for AWE, and only histopathological examination of the specimen can confirm the diagnosis. However, if a painful nodular mass is located adjacent to a surgical scar in a female patient, AWE should be a leading consideration in the differential diagnosis, along with suture granuloma in cases of old surgical scars.

## Figures and Tables

**Figure 1:**
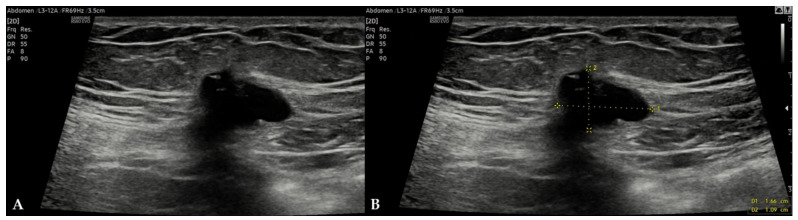
AWE on B-mode US: (**A**) irregularly shaped, hypoechoic mass, with lobulated and partially indistinct margins, posterior shadowing, and hyperechoic rim surrounding the mass. Within the mass, a few linear hyperechoic structures could also be seen; (**B**) mass dimensions were 1.7 × 1.1 cm.

**Figure 2:**
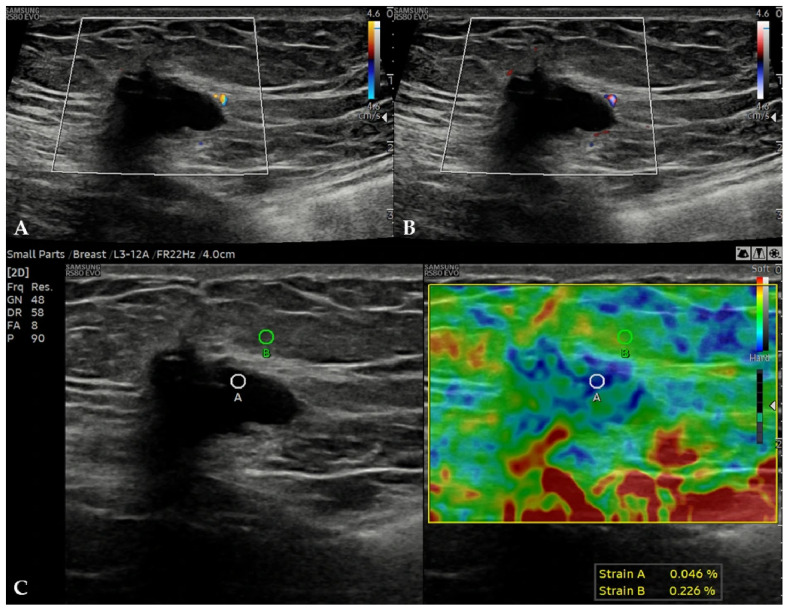
AWE on color and power Doppler and strain elastography: (**A**,**B**) mass showed mild rim-like vascularization on color and power Doppler, respectively; (**C**) mass was predominantly soft, with several areas of increased stiffness in the center and at the periphery. The strain ratio of stiffer areas was 4.91.

**Figure 3:**
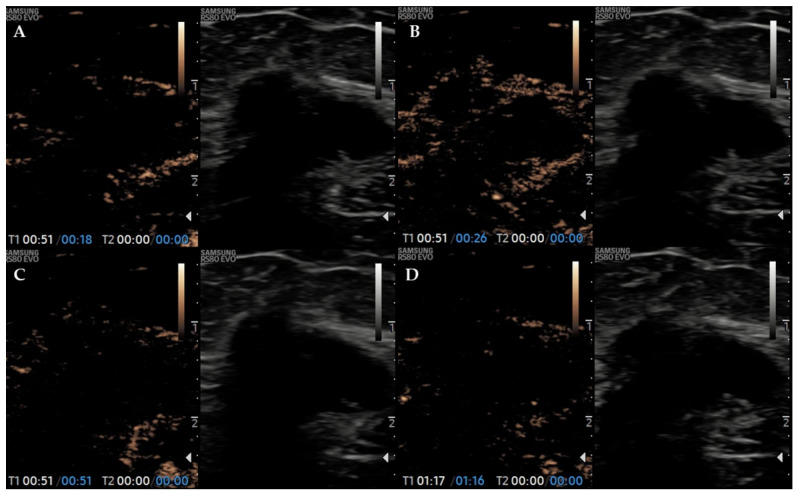
AWE on CEUS (**A**–**D**): Centrally non-enhancing mass with rapid, progressive, rim-like enhancement within the first 30 s of evaluation. Total scan time was 01:17 min.

**Figure 4:**
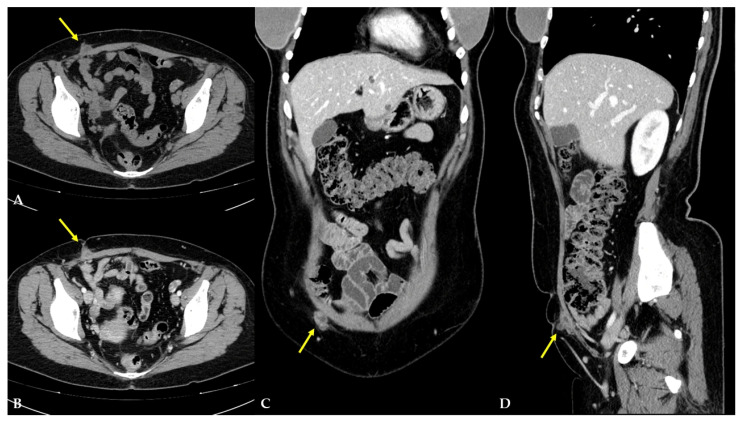
AWE on CT (yellow arrow): (**A**) axial plane, non-contrast-enhanced; (**B**) axial plane, contrast-enhanced; (**C**) coronal plane, contrast-enhanced; (**D**) sagittal plane, contrast-enhanced.

**Figure 5:**
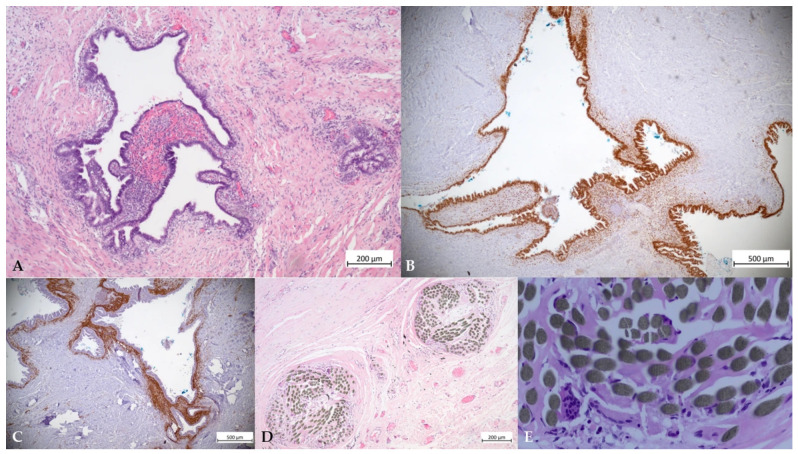
Histopathological analysis of AWE: (**A**) H&E staining, ×100 magnification, endometrioid glands surrounded by endometrial-type stroma and extravasated red blood cells, consistent with a diagnosis of endometriosis; (**B**) ER immunostaining, ×50 magnification, estrogen receptor (ER) shows strong nuclear staining in endometrial glandular epithelium; (**C**) CD10 immunostaining, ×50 magnification, CD10 shows cytoplasmic staining in endometrial stromal cells; (**D**) H&E, ×100 magnification, suture material with foreign-body-type giant cell reaction; (**E**) H&E staining, ×400 magnification, multinucleated giant cells surrounding the suture fibrils.

**Table 1:** List of reported cases of AWE in the appendectomy scar.

N	Year	Age	Parity	Endometriosis History	Clinical Presentation	Latency	Imaging	Treatment	Recurrence	Ref.
1	1957	43	0	No	Cyclic pain in the appendectomy scar with the development of a palpable mass and a cutaneous fistula	34 years	No	Wide surgical excision, no mesh	N/A	Haser [7]
2	1976	41	2	No	Cyclic pain in the appendectomy scar with the development of a palpable mass	35 years	No	Wide surgical excision (peritoneal penetration, contact with cecum), no mesh	No	Hofmann et al. [8]
3	1980	17	0	No	Cyclic pain and swelling of the appendectomy scar with the development of a palpable mass	2.5 years	No	Local surgical excision, no mesh	No	Song et al. [9]
4	1999	28	0	No	Cyclic pain in the appendectomy scar with the development of a palpable mass and black blood discharge	26 years	No	Wide surgical excision (peritoneal involvement), mesh placement	No	Lamblin et al. [10]
5	2010	37	2	Yes	Cyclic painful mass (right trocar tract)	8 months	No	Local surgical excision, no mesh	Yes, wide surgical excision, mesh placement	Akbulut et al. [11]
6	2012	33	2	Yes	Cyclic pain in the appendectomy scar with the development of a palpable mass	19 years	CXR, AXR, Abdominal CT	Local surgical excision, no mesh	No	Mizutani et al. [12]
7	2018	36	1	Yes	Non-cyclic, progressive worsening of the pain in the appendectomy scar with a palpable mass	7 years	Abdominal US and CT	Local surgical excision, no mesh	N/A	Amini et al. [13]
8	2022	35	3	No	Cyclic pain and swelling of the appendectomy scar	27 years	No	Local surgical excision, no mesh	No	Ahmad et al. [14]
9	2022	29	2	No	Cyclic pain and swelling of the appendectomy scar	2 years	Abdominal US and MRI	Wide surgical excision (peritoneal involvement), no mesh	No	Kala et al. [15]

N/A—unknown; CXR—chest X-ray; AXR—abdominal X-ray; CT—computed tomography; US—ultrasound; MRI—magnetic resonance imaging.

## Data Availability

Data are contained within the article, and further inquiries can be directed to the corresponding author.
